# Increase in the prevalence of arthritis in adulthood among adults exposed to Chinese famine of 1959 to 1961 during childhood: A cross-sectional survey

**DOI:** 10.1097/MD.0000000000006496

**Published:** 2017-03-31

**Authors:** Xianglong Xu, Lingli Liu, Wenxi Xie, Yong Zhang, Huan Zeng, Fan Zhang, Cesar Reis, Xianqing Cao, Yong Zhao

**Affiliations:** a School of Public Health and Management; b Research Center for Medicine and Social Development; c The Innovation Center for Social Risk Governance in Health; d School of the Second Clinical, Chongqing Medical University, Chongqing, China; e Department of Preventive Medicine, Loma Linda University Medical Center, CA.

**Keywords:** adulthood, arthritis, Chinese famine, early life, malnutrition

## Abstract

The developmental origins hypothesis postulates that under-nutrition in the early stage of life is associated with an increased risk of disease in adulthood. This study aimed to examine the association of exposure to the Chinese famine of 1959 to 1961 in early life with the risk of arthritis in adulthood.

From July to September 2009, the study adopted multistage stratified random sampling cross-sectional survey to recruit 1224 eligible adults in Chongqing. Famine exposure groups were categorized into 3 groups: (1) childhood exposure, (2) fetal exposure, and (3) nonexposure. Self-reported arthritis of physician diagnosis was obtained. A total of 1224 eligible respondents were interviewed, including 299 individuals exposed during childhood, 455 exposed when fetal, and 470 without exposure.

The prevalence of arthritis in adulthood among individuals exposed to famine during childhood was significantly higher than those not exposed (17.39% vs 11.28%, odds ratio [OR] = 1.573 with a 95% confidence interval of [CI] [1.020, 2.424]). Persons exposed to famine during the fetal period did not significantly contribute to a higher rate of arthritis in adulthood than those who were not exposed to famine (13.19% vs 11.28%, OR = 1.072, 95% CI = 0.713, 1.613). In addition, education level, the average monthly income, sleep status, and satisfaction of the present living condition were associated with the risk of arthritis in adulthood.

Exposure to the Chinese famine during childhood may be associated with an increased risk of arthritis in adulthood. This study suggests that early life nutrition may have an effect on the risk of arthritis in adulthood.

## 1 Introduction

Arthritis is a common disease worldwide. The overall prevalence of arthritis among middle-aged and older adults was 31.4% in China.^[1]^ The prevalence of rheumatoid arthritis varies between 0.3% and 1% and is more prevalent in developed countries.^[2]^ About 22.7% of American adults, or 52.5 million people aged ≥18 years in the civilian, noninstitutionalized population, have self-reported doctor-diagnosed arthritis.^[3]^ A study estimated that compared to 52.5 million adults in 2010 to 2012,^[3]^ an estimated 67 million (25% of the projected total adult population) adults aged ≥18 years will have doctor-diagnosed arthritis in America by the year 2030.^[4]^ Owing to its increasing prevalence and association with cardiovascular disease, which is the leading cause of death in industrialized countries, it has become an important public health problem.^[5]^

In contrast to the Dutch and Leningrad famines, Chinese famine of 1959 to 1961 occurred more recently, had a longer duration (3 years), was not caused by war, affected most areas of China, and had serious consequences. The Chinese famine that occurred between 1959 and 1961 is the largest food crisis registered in human history and has resulted in ∼30 million fatalities.^[6]^ Historical records reveal that this famine stretched out to Chongqing city. Before the famine, China's grain output continued to grow and reached a peak of 200 million tons in 1958.^[7]^ In 1959, grain output fell by 15%, whereas the output continued to decline in the next 2 years, only reaching ∼70% of the 1958 record level.^[7]^ In 1962, the declining trend in grain production stopped, and the total grain production did not recovered to the 1958 output level until 1966.^[8]^ During 1959 to 1961, estimated daily per capita availability of food energy decreased significantly, far below average food energy requirements, and the worst food accessibility rate was in 1960 (only 1500 calories daily).^[9]^ Compared with the national average death rate was ∼11 per thousand in 1956 to 1958, the death rates were 14 per thousand in 1959, 25 per thousand in 1960, and 14 per thousand in1961, and fertility rates also dropped sharply in 1959 to 1961.^[10]^ Chinese famine of 1959 to 1961 can provide a quasi-experimental setting for research on the long-term effects of malnutrition on adult chronic diseases.

The developmental origins hypothesis proposes that malnutrition in early life is associated with an increased risk of disease in adulthood.^[11]^ The adverse long-term consequences of famine exposure during early life may be augmented in a nutritionally rich environment in later life.^[12]^ An early-life exposure to famine is associated with an elevated risk of having diabetes,^[13]^ metabolic syndromes,^[14]^ hypertension,^[15]^ short height,^[8]^ and excessive weight^[16]^ in adulthood. Metabolic triggered inflammation is mainly caused by nutrient overload and metabolic surplus.^[17]^ Based on these findings, this study assumed that there is an association between exposures to the Chinese famine in early life and the risk of arthritis in adulthood.

Certain factors have been found to be associated with the risk of arthritis, including gender, race, age, occupation, physical activity, smoking, and obesity.^[3,18–21]^ To our knowledge, presently there is no study showing the relationship between early exposure to famine and occurrence of arthritis in adulthood. Factors associated with arthritis of adults have not been sufficiently clarified. Elucidation of such factors leading to arthritis in adulthood would be helpful to reduce the prevalence of such public health concern. In view of this, this study aimed to analyze exposure to the Chinese famine in early life and the possible effects on arthritis in adulthood. The assumption is that experiencing fetal or childhood exposure to the Chinese famine in early life leads to an increased risk of arthritis in adulthood.

## 2 Methods

This study was prepared according to the strengthening the reporting of Observational Studies in Epidemiology (STROBE) Statement.

### 2.1 Study design

This study design and participants have been reported previously.^[22,23]^ The cross-sectional survey was conducted in Chongqing city in July 2009. This cross-sectional study used 3-stage stratified random sampling method to recruit participants. The survey was conducted in compliance with the Ethical Committee of Chongqing Medical University. All study participants gave informed written consent.

### 2.2 Participants

This study used 3-stage stratified random sampling method to recruit participants. Eligible participants were those born between 1956 and 1964 and were aged 45 to 53 years old during time of the survey. Firstly, about 10 districts and counties were randomly selected in Chongqing. Secondly, about 8 to 10 villages were selected in each selected district or county. Finally, about 10 to 15 participants were randomly selected in selected each village. Eligibility criteria were: born in the years from 1956 to 1964, born in Chongqing. Participants should be excluded who met the following exclusion criteria: cognitive disorders, including amnesia, dementia, and delirium.

### 2.3 Study size

According to the literature,^[1]^ the prevalence of arthritis among middle-aged and older Chinese adults was 31.4%. We set the probability of arthritis *P* = 0.314. (*P* = 0.314;*Q* = 1 − *P* = 1 − 0.314 = 0.686 margin of error *d* = 0.1, *P* = 0.1 × 0.314 = 0.0314, *Z*_α_ = 1.96; the sampling size  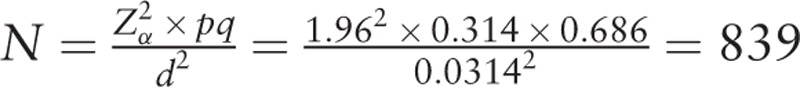). A total of 1224 eligible respondents were included in the analysis.

### 2.4 Variables and measurement

#### 2.4.1 Socio-demographic variables

Demographic data included gender, date of birth, job status (employed, unemployed, and retired), education level (basic education equal to/under junior middle school; secondary education equal to/over senior high school [including vocational/technical secondary school and junior college]; higher education equal to/over senior college), method of delivery (natural birth/cesarean delivery/ not aware), feeding procedure (breastfeeding/artificial feeding/ mixed feeding), marital status (unmarried/married or cohabitation/divorce or separated/widowed), and average monthly income (<¥850 /¥850– ¥1600 />¥1601, (1 USD = 6.86 ¥ in February 2017).

#### 2.4.2 Chinese famine exposure status variables

The subjects were divided into 3 groups by the birth years: (1) childhood exposure group: the pre-famine cohort (1956–1958), (2) fetal exposure group: the famine cohort (1959–1961), and (3) nonexposure group: the post-famine cohort (1962–1964). This classification can be seen in a previous published Chinese famine study.^[24]^

### 2.5 Lifestyle variables

Lifestyle questions involved the following: smoking (yes/ no), alcohol drinker (yes/ no), do you have a regular physical activity (never or seldom/ sometimes/ regularly), self-reported appetite status (good/ average/ poor), do you have a regular life routine (usually/ sometimes/ seldom), self-reported sleep status (good/ average /poor), condition of getting along with family (harmony/ average/ poor), condition of getting along with colleague or friend (harmony/ average/ poor), and present living conditions (satisfied/ average/not satisfied/). Participants were classified into low-, normal-overweight and obesity-BMI groups as follows: low (BMI <18.5), normal (18.5≤ BMI <24), overweight (24≤ BMI <28), and obesity (BMI ≥28).^[25]^

### 2.6 Outcome variables

Arthritis status was categorized into arthritis and nonarthritis. Arthritis patients were self-reported previous diagnosis of arthritis. We asked all respondents whether they had self-reported previous diagnosis of arthritis, including osteoarthritis, rheumatoid arthritis, spondyloarthritis, etc. There were no exclusion criteria regarding type of arthritis, which meant that any type was categorized as a yes in the study.

### 2.7 Bias

To reduce potential sources of bias, the following measures were implemented. First, face-to-face interview was conducted by senior medical school students. Those senior medical school students underwent standardized training and were familiar with the objectives and methodology of the study. Second, the questionnaires were designed by many experts in nutrition, health behavior and health promotion, and epidemiological background to make certain of its validation. What is more, a pilot test was conducted. Participants were interviewed in private using a pre-tested structured questionnaire. A total of 40 individuals participated in the pilot study. Questionnaire was modified based on the results of the pilot study, especially the description of questions and the answer options.

### 2.8 Statistical methods

The chi-square test was utilized to compare the differences between arthritis and nonarthritis. Logistic regression analysis was utilized to explore the factors associated with arthritis in adulthood. The dependent variable “arthritis status” was recorded into 2 categories: 1 = “arthritis status” and 0 = “not arthritis status”. In the logistic regression model, odds ratio (OR) and 95% confidence intervals (CI) were calculated. In logistic regression model 1, timing of exposure to famine was considered in modeling the determinants that affect arthritis in adulthood. In logistic regression model 2, timing of exposure to famine, gender, marital status, smoking, alcohol drinking, condition of getting along with family, condition of getting along with a colleague or friend, and present living conditions were considered in modeling the determinants that affect arthritis in adulthood. The multivariate model was statistically significant in the model coefficient test (*P* <0.05), and reach a good fit in Hosmer and Lemeshow test (*P* = 0.09). All statistics were performed using 2-sided tests, and statistical significance was pre-defined as *P* <0.05. All data analyses were performed using statistical software (SAS version 9.1; SAS Institute, Cary, NC).

## 3 Results

A total of 1224 eligible respondents were investigated, including 165 (13.48%) arthritis and 1059 (86.52%) nonarthritis. Among the 165 arthritis, 36.36% had fetal famine exposure, 31.52% had childhood famine exposure, and 32.12% had no-exposure. Statistically significant differences were observed between the arthritis groups and nonarthritis groups on the variables: Education level (*P* <0.0001), average monthly income (*P* <0.0001), appetite status (*P* <0.0001), sleeping status (*P* <0.0001), and condition of getting along with the family (*P* <0.0001). However, no statistical significant differences were observed on the variables: the timing of exposure to famine, gender, marital status, job conditions, BMI group**, s**moking, alcohol drinker, regular physical activity, regular daily life, condition of getting along with colleague or friend, mode of delivery, and feeding procedure between the arthritis groups and nonarthritis groups (Table 1).

**Table 1:**
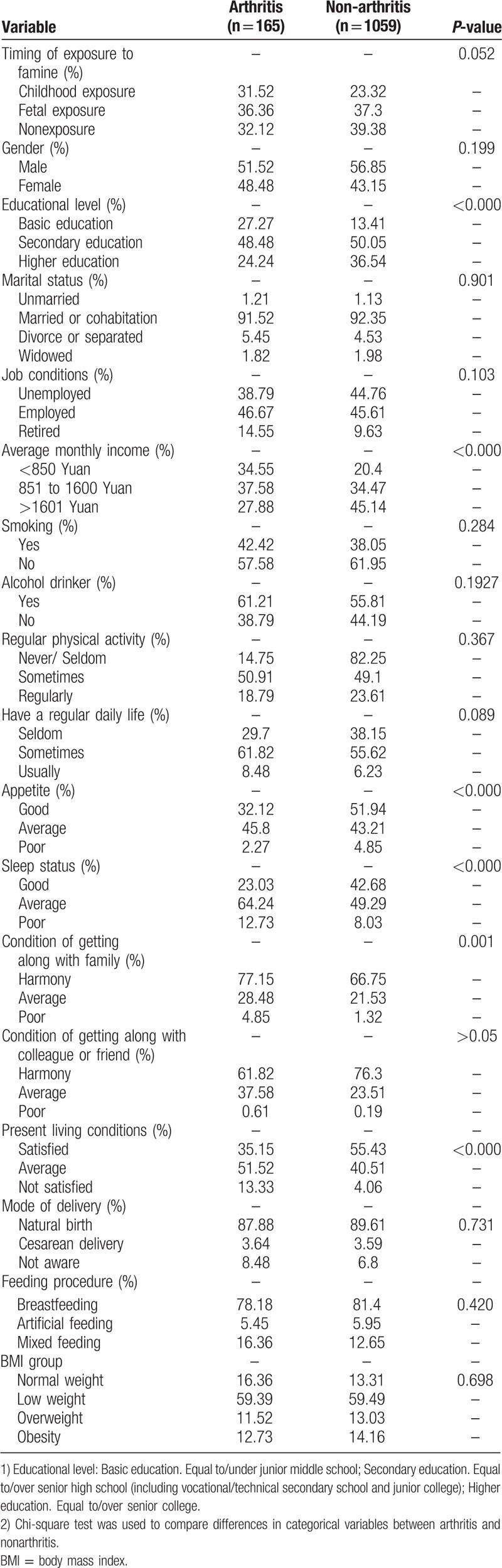
Characteristics of the study participants by arthritis, Chongqing, China (n = 1224).

The multivariable logistic regression model showed the association of famine exposure with the risk of arthritis in adulthood. In model 2, the prevalence of arthritis in adulthood among individuals exposed to famine during childhood was significantly higher than those not exposed (OR = 1.573, 95% CI = 1.020, 2.424). However, people exposed to famine during the fetal period were not significantly in the prevalence of arthritis in adulthood than those who were not exposed to famine (OR = 1.072, 95% CI = 0.713, 1.613; Table 2).

**Table 2:**
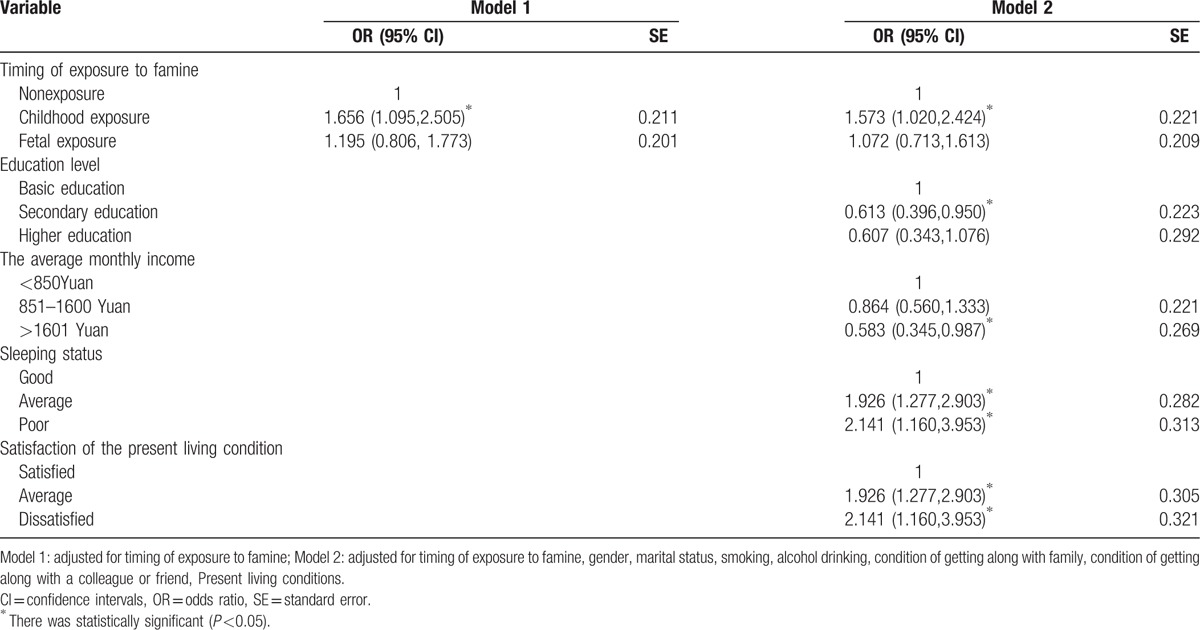
Odds ratios (95% CI) for arthritis in adulthood, Chongqing, China (n = 1224).

The multivariable logistic regression model also showed the association of sociodemographic factors and lifestyle factors associated with the risk of arthritis in adulthood. In model 2, comparing with basic education level, those with secondary level were less likely to have arthritis (OR = 0.613, 95% CI = 0.396, 0.950). People with income >1601 Yuan were less likely to have arthritis than those with <850Yuan (OR = 0.583, 95% CI = 0.345, 0.987). Respondents with average sleep quality were more likely to have arthritis than those with good sleep quality (OR = 1.926, 95% CI = 1.277, 2.903). Also those with poor sleep quality are more likely to have arthritis than people with good sleep quality (OR = 2.141, 95% CI = 1.160, 3.953). People with average satisfaction of the present living conditions are more likely to have arthritis than those satisfied with the present living condition (OR = 1.926, 95% CI = 1.277, 2.903). The dissatisfied ones with the present living condition are more likely to have arthritis than the satisfied ones with the present living condition (OR = 2.141, 95% CI = 1.160, 3.953; Table 2).

## 4 Discussion

### 4.1 Primary findings

Approximately 13.48% of the respondents self-reported their diagnoses of arthritis in adulthood. Among the arthritis participants, 36.36% had fetal famine exposure, 31.52% had childhood famine exposure, and 32.12% had no-exposure. People exposed to famine during childhood were more likely to have arthritis than nonexposed to famine. However, people exposed to famine during the fetal period did not significantly contribute to a higher rate of arthritis in adulthood than those who were not exposed to famine.

### 4.2 Implications for physicians, policymakers, and future research

This study found that compared with nonexposed, childhood exposure to famine was associated with an increased risk of arthritis in adulthood. A previous study found that people who experienced malnutrition during early life and a nutritionally rich environment in later life have a higher risk of developing metabolic syndrome because of metabolic maladjustment.^[14]^ Metabolic imbalances are more likely to induce overnutrition-related disease, such as atherosclerotic vascular disease and type 2 diabetes. In addition, metabolic triggers inflammation that may be associated with arthritis. Metabolic-triggered inflammation caused by nutrient overload and metabolic surplus consists of components (such as abnormal metabolites, pro-inflammatory cytokines and adipokines, acute phase proteins, vitamin D deficiency, deregulated microRNAs, and obesity) that may play a role in the pathophysiology of osteoarthritis.^[26]^ A previous study also demonstrated the pathological hallmark of osteoarthritis: osteoarthritis is part of a generalized metabolic disorder in which varieties of interrelated metabolic and hemodynamic factors conduce to the progressive degradation of articular cartilage.^[5]^ These findings may further confirm that early-life exposure (especially childhood exposure to famine) affects health conditions in adulthood. Those adults who were undernourished in their early life may have a higher tendency to get arthritis in adulthood. Such analysis can contribute to a new direction for the diagnosis and prevention of arthritis; for instance, strategies targeting early malnutrition, especially during childhood, may be effective in preventing arthritis. Furthermore, this study may confirm the developmental origins hypothesis that early malnutrition is associated with diseases in adulthood. This study also provides some implications for future studies of the risk factors for arthritis. However, additional investigation should be done to corroborate the present findings.

This study found that respondents who were with low education level and lower income were not satisfied with their present living conditions and those who had poor sleep quality were associated with an increased risk of arthritis in adulthood. A previous study found that low socioeconomic status was associated with an increased risk of arthritis, education level was also one of the independent factors along with fatigue, and fatigue had a great influence on rheumatoid arthritis patients.^[27]^ Previous studies found that 40% to 80% of rheumatoid arthritis patients reported significant fatigue, and fatigue was their most frequently disabling symptom.^[28,29]^ This study found that poor sleep quality was associated with the risk of arthritis. A previous study also reported that poorly controlled rheumatoid arthritis can reduce sleep quality.^[30]^ Furthermore, low life satisfaction was also associated with arthritis. A possible reason for that is those experiencing low life satisfaction with the present living condition are more likely to have poor quality of life. And dissatisfaction may have an effect on immunity and for that reason leads to autoimmune diseases. Moreover, poor quality of life may have an adverse impact on arthritis.^[31]^ Arthritis patients were less likely to reach the recommended levels of moderate or vigorous physical activity than adults without arthritis, and ∼37% of arthritis patients were inactive.^[20]^ Thus, it is necessary to focus on low socioeconomic status populations to control the prevalence of arthritis.

### 4.3 Strengths and limitations of the study

Based on our knowledge, this is the first study to examine the association of exposure to the Chinese famine of 1959 to 1961 in early life with the risk of arthritis in adulthood. This study suggests that childhood exposure to the famine may contribute to a higher rate of arthritis in adulthood. This study can provide some insights on possible interventions to prevent and control arthritis.

The present study also has certain limitations that should be noted. First, age and cohort/ famine exposure may be confounded factors. This study used a range of birth year cohorts that included the pre-famine cohort (before the famine), fetal exposure cohort (during the famine), and nonexposure cohort (after the famine). Previous findings showed that this approach could provide a way to explore a range of age exposures and the various effects of these factors.^[15]^ However, in the future, a comparison group from another similar province that was not affected by the famine would empower us to make a causal claim. Second, this is an exploratory study, and self-reported previous diagnosis of arthritis was obtained, not confirmed directly with the doctors’ diagnosis. This self-reported diagnosis may not be accurate, and some participants with arthritis do not clearly know that they have the disease, especially in rural areas in China. Self-reported arthritis may underestimate the prevalence of arthritis. To reduce bias, for each participant that reported arthritis, the investigator further confirmed the condition. Third, this is a cross-sectional analysis, which reduces the ability of the researchers to assume direct causal inferences. We recognize that unmeasured variables are underestimated in this study, and their effect may better explain the observed relationships and determine the direction of causality. Fourth, the participants are middle aged (45–53 years old), and the findings may not apply to the younger or older. Fifth, this study did not distinguish among types of arthritis, for example osteoarthritis and rheumatoid arthritis. Furthermore, a study in 2008 showed that the prevalence of rheumatoid arthritis is quite low and <1%.^[32]^ If this prevalence holds true in our sample, from a total of 1224 patients reported in the study, we would have only 12 patients with rheumatoid arthritis. Conclusions of the study related to rheumatoid arthritis patients should be made cautiously. This study probed whether exposure to famine in early life was associated with arthritis, and we only attempted to provide some insights for future studies. For instance, the association of exposure to the Chinese famine of 1959 to 1961 in early life with different specific types of arthritis would be a subsequent step for further in-depth study. Sixth, this study used a proxy measure for malnutrition. Famine exposure groups were categorized into 3 groups based on birth date. Potentially, not all respondents’ families were equally affected by the famine. We did not measure the degree of exposure because it would be a very difficult criterion to estimate for the scope of this study. Seventh, inability to use proper statistical techniques (GEE/random effects logistic model) to adjust for potential intracluster correlation because of lack of suitable indicator variable(s) is a limitation. Because the strength of association between the primary exposure (exposure to famine) and outcome variable (arthritis status) is weak (the lower boundary of the 95% CI for childhood exposure to famine was 1.02), intracluster correlation may undermine the study findings and alter the conclusions. Although this limitation may exist, this study also provides some clues for future studies of the risk factors for arthritis. This may indicate that further studies are needed about the association. Further studies may adjust for potential intracluster correlation to further test. Finally, the lifestyle instrument has not been tested for its psychometric properties.

## 5 Conclusion

Approximately 13.48% of the respondents self-reported their diagnoses of arthritis in adulthood. These findings suggest that childhood exposure to the famine may contribute to a higher rate of arthritis in adulthood. Consequently, nutritional status and health during childhood need more attention. Further studies will be required to test the association of exposure to the Chinese famine of 1959 to 1961 in childhood with an increased risk of arthritis in adulthood.
